# Collectanea Medica: Consisting of Anecdotes, Facts, Extracts, Illustrations, &c.

**Published:** 1826-01

**Authors:** 


### COLLECTANEA MEDICA:

CONSISTING OF

### ANECDOTES, FACTS, EXTRACTS, ILLUSTRATIONS, &c.

***Relating to the History or the Art of Medicine, and the Collateral Sciences.***

---

Floriferus, ut apes, in saltibus omnia libant,

Omnia nos, itidem, depascimur aurea dicta.

---

### ART. I.—*Of* DR. BAILLIE's *Experience in Fevers.* [From his "*Lectures and Observations in Medicine.*"]

WHILE I was a physician of St. George's Hospital, which was during a period of thirteen years, I saw a good many cases of typhous fever. There were generally three or four cases of such fevers under my care at a time. Since I have ceased being a physician to that hospital, and more especially since my patients have been chiefly in the upper ranks of society, I have not seen more than three or four of such fevers in a twelvemonth. With respect to the contagious nature of these fevers, I am convinced that it is in general not considerable. I do not recollect an instance in which a patient in that hospital communicated the infection to a patient lying in the next bed. When patients are crowded together, and the apartments are ill ventilated, I entertain no doubt of this species of fever being capable of being communicated readily from one individual to another.

These fevers are sometimes without any symptoms which denote a local affection of a vital organ, but very frequently there are symptoms which indicate an inflammatory action of some of the viscera in the chest or belly, or of the brain.

In these fevers, I have met with no remedies which possess any specific powers of cure, or which are capable of shortening, in any material degree, their duration. Before they are fully formed, they are sometimes cut short by an emetic, by active purgatives, by profuse perspiration, or by cold affusion ; but, when they are quite established, I do not recollect that I have seen any instance in which they have been shortened by these means. The most successful method of treating these fevers, as far as I have seen, is to remove or mitigate the symptoms as they arise. The symptoms denoting an affection of the brain should be relieved as speedily as possible by cupping, leeches, and the application of cold to the head. Cloths dipped in iced water, and kept almost constantly applied to the shaved scalp, have appeared to me more effectual in removing delirium than any other remedy.

When there is pain in any part of the chest, or difficulty of breathing, these symptoms should be relieved as soon as possible by cupping or leeches, or blisters, and by saline medicines.

If there be any pain in the abdomen, or any symptoms denoting an affection of the liver, the stomach, and the bowels, these are to be relieved by their appropriate remedies.

If there be too vigorous a circulation over the body, without any apparent local affection, it may be corrected by a very cautious bleeding from the arm, by purging, and by saline medicines. If the actions of the constitution be feeble, they may be strengthened by tonic and stimulating remedies, the best of which I believe to be wine in suitable doses. By this mode of treatment, fevers will often terminate favourably, which otherwise would have been fatal.

During the greater part of the time in which I have practised medicine, physicians in general, and myself among that number, have, I believe, been too sparing in taking away blood in typhous fever. It was hardly ever directed to be taken away from the arm, and not often locally, except by the application of leeches to the head. Of late years many physicians have gone into the opposite extreme, and have taken away blood too profusely. In the course of a few years this remedy, like every other, will find its proper level. During the course of a fever, patients require but little nourishment; and this should in a great measure consist of farinaceous matter. Even when the fever has entirely subsided, animal food should be taken, for some time, very cautiously and sparingly. I have known some instances of the most serious relapses of fever, from patients having taken animal food too soon and in too large quantity; and I am disposed to think that the greater number of relapses arise from this cause.

*Of Intermittent Fevers.*—I have always practised in London, and have therefore not had many cases of intermittent fever under my care. While I was a physician of St. George's Hospital, I perhaps saw five or six cases of it in a year; and this chiefly occurred among the poor Irish who lived or lodged in St. Giles's. In some of these cases, the origin of the disease could be clearly traced to marshy effluvia; but in others this cause could not be traced, as the patients reported that they had lived in St. Giles's for several years, and had always been employed as labourers in London. They may, however, have been exposed to marshy effluvia in the neighbourhood of London, without knowing or recollecting it.

I have known a good many cases in which bark alone would not cure an ague. In all of these cases, as far as I now recollect, when a grain of calomel was given every night for eight or ten nights, bark cured the ague in the course of a few days. This practice I learnt from my friend Dr. David Pitcairn. The powder of bark I consider as a more efficacious remedy for agues than the extract of bark.

According to my experience, arsenic cures agues in general sooner than bark, and produces no bad effect, if it be given in proper doses, and be not continued too long. When the ague has been stopped for three or four days, the arsenic should be given up, and half a drachm of bark, in powder, should be given three or four times a-day, for perhaps a week.

I have known some cases of ague cured by the powder of calamus aromaticus; and I have understood that it is not an uncommon remedy among the lower orders of people in Sussex. 1

**Art. II.**—***An extraordinary Case in which Clots of Blood oozed from the Face of a Girl.*** **Communicated to Dr. Chapman. [From the *Philadelphia Journal,* No. 2, New Series.]**

Sir,—In the sixteenth Number of your Journal, you express a wish to have an authentic account of the extraordinary case of a girl in this neighbourhood, thirteen years old, "from whose face and under the eye clots of blood would occasionally ooze."

As the case fell under my observation, I have presumed to give you the following facts.

The accounts of the case by General Eavans, Dr. Shuler, and Drs. Smith and Casey, some time ago in the Indiana papers, are correct and without exaggeration, as can be testified by a hundred people.

The girl is small of her age, but well made, of a good disposition, and of a poor but honest and industrious family. In the spring and summer of 1823, a little blood would occasionally appear about her eye and face, which neither excited alarm nor curiosity. In the November following, however, the quantity became suddenly very large. The family in which she lived think a pound was found some mornings about her face and pillow in clots. When this oozing of blood had continued about five days, a physician being called in applied, without knowing for what reason, a plaster to the place from whence the hemorrhage came. But the blood soon pushed it off. About this time, fimbriated substances, resembling moss or spiders, and pieces of bone, were discovered among the clots. Henceforward things changed.

Generally a single clot of blood would appear first in the morning, and be followed by lumps of fleshy substance and pieces of bone, alternately, all day. The bones were of every size under the weight of two drachms, of various and irregular shapes, generally having one or two smooth sides without any periosteum, while the other sides appeared much like fractured surfaces. They continued to come for about six weeks, with an intermission of three or four days. At first they came as often as once in every ten or fifteen minutes, and were very hard; at last they came much more seldom, and gradually assumed a cartilaginous appearance. They generally adhered to the cheek slightly, by a kind of glutinous substance, till pulled off. Their first appearance was astonishing. While the bystanders would be gazing at the place from whence they came, they would be suddenly surprised with their appearance, without being able to tell how they came; for the girl would feel no pain, and not the least sign of an opening or a scratch could ever be discovered. All agreed in thinking they must come through the skin.

The lumps of flesh that alternated with the bones, were about the size of the end of a finger, and had much the appearance of the soft kind of polypus.

All these things appeared about the left eye, and mostly upon the cheek under the eye. Two pieces of cartilage made their appearance between the eyelid and eye. The last of all that appeared was a cartilage nearly as large as the nose, which came through the left nostril, causing in its passage considerable irritation.

During this extraordinary case, I visited the girl several times, and once in company with several other physicians. Not the least sign of swelling, discoloration, soreness, or any thing else indicating disease, could ever be discovered about the girl; except she acknowledged that she had occasionally a slight pain about her hips and loins.

I have been told that, soon after the last cartilage made its appearance, she commenced menstruating regularly, and nothing strange has ever appeared about her since. During the time the bones were coming, the girl was subject to eructating, after eating, a kind of gas of a very strong smell, which the family compared to that of whiskey.

---

